# Supplementary material for: Chromothripsis during telomere crisis is independent of NHEJ, and consistent with a replicative origin
Source: Genome Res. 2019 May;29(5):737–49. doi: 10.1101/gr.240705.118 (PMC6499312; doi:10.1101/gr.240705.118)
Supplement: Supplemental Material [file supp_gr.240705.118_Supplemental_file_1.zip › contigs/annotated_contigs/DB55/contig.2.DB55_length_264_mean_cov_2.27272727273.docx]

**DB55_length_264_mean_cov_2.27272727273**

```
CTGGACAACATGACAAAACCCTGTATTCACACACAAAAATACATTAGCTGGGTGTGGTGGTGTGCACCTGTAGTCCTAGATATTCGGGA
 >chr9:17020744-17020898 - E=1e-78
GACTGAGGTGGAAGGATCACTTGAGCCAGGGAGGTCAAGGCTGCAGTGAGCTATCATTGCGC|TAC|CTAGGTGATAGATTGATAGGTG
                                                               >chr9:17281278-17281391
CTGCAAACCACCATGGCACATATTTACCTACATAACAAACCTGCACATCCTGCACGTGTATCCTGGAACTTGAAACAAAATTAATTTT- E=8e-57
```
